# Mindfulness-Based Cognitive Therapy and the Adult ADHD Brain: A Neuropsychotherapeutic Perspective

**DOI:** 10.3389/fpsyt.2016.00117

**Published:** 2016-06-27

**Authors:** Katharina Bachmann, Alexandra P. Lam, Alexandra Philipsen

**Affiliations:** ^1^School of Medicine & Health Sciences, University of Oldenburg, Oldenburg, Germany; ^2^Psychiatry and Psychotherapy, University Hospital, Karl-Jaspers-Klinik, Bad Zwischenahn, Germany; ^3^Department of Psychiatry and Psychotherapy, University Medical Center Freiburg, University of Freiburg, Freiburg, Germany

**Keywords:** neuropsychotherapy, ADHD, adult, MBCT, non-psychopharmacological treatment

## Abstract

Attention-deficit/hyperactivity disorder (ADHD) is a recognized serious mental disorder that often persists into adulthood. The symptoms and impairments associated with ADHD often cause significant mental suffering in affected individuals. ADHD has been associated with abnormal neuronal activity in various neuronal circuits, such as the dorsofrontostriatal, orbitofrontostriatal, and frontocerebellar circuits. Psychopharmacological treatment with methylphenidate hydrochloride is recommended as the first-line treatment for ADHD. It is assumed that medication ameliorates ADHD symptoms by improving the functioning of the brain areas affected in the condition. However, side effects, contraindications, or non-response can limit the effectiveness of a psychopharmacological treatment for ADHD. It is therefore necessary to develop non-pharmacological interventions that target neuronal mechanisms associated with the condition in the same way as pharmacological treatment. We think that mindfulness meditation employed as a neuropsychotherapeutic intervention could help patients with ADHD to regulate impaired brain functioning and thereby reduce ADHD symptoms. In this paper, we highlight the mechanisms of such mindfulness meditation, and thus provide a rationale for further research and treatment development from a neuropsychotherapeutic perspective. We conclude that mindfulness meditation employed as a neuropsychotherapeutic intervention in therapy is a promising treatment approach in ADHD.

## Current State of Treatment of ADHD in Adulthood

Attention deficit/hyperactivity disorder (ADHD) is a serious mental disorder characterized by three core symptoms: inattention, impulsivity, and hyperactivity. In up to 60% of cases, ADHD symptoms persist into adulthood (1). It has been estimated that about 3.4% of the adult population is affected by ADHD (2). The clinical picture of the condition is quite heterogeneous with respect to the expression and severity of symptoms, as well as its pathogenesis (3, 4).

It is assumed that the disorder relies strongly on impairments to neurobiological function (5, 6). Individuals with ADHD show abnormal neuronal activity in dorsofrontostriatal, orbitofrontostriatal, and frontocerebellar circuits (6). Furthermore, abnormal functional connectivity in the default-mode network (DMN) has been suggested (7).

Adults with ADHD often suffer from comorbid disorders (e.g., depression or anxiety disorders) and negative psychosocial consequences (8). Therefore, a multimodal treatment approach that takes into consideration both the ADHD and the comorbid disorders and psychosocial functioning is currently the gold standard in the treatment of adult ADHD (9).

Psychopharmacological treatment with methylphenidate hydrochloride is recommended as the first-line treatment for ADHD core symptoms (9). Methylphenidate influences dopaminergic and noradrenergic systems of the striatum, prefrontal cortex, locus coeruleus, and somatosensory cortex. Dopamine plays an important role in drive and motivation, and noradrenalin in attentional processes (10–12). Therefore, it has been suggested that the positive effects of methylphenidate on ADHD can be attributed to improving the functioning of brain areas involved in attentional and motivational processes (13). These positive effects can be augmented when combined with individual or group cognitive behavioral therapy (14).

However, while psychopharmacological treatment with methylphenidate has undoubted positive effects on ADHD symptoms, it also has significant limitations. About 20–50% of adult ADHD patients are non-responders (15). Also, a study by Tucha et al. (16) showed that although methylphenidate reduced deficits in attentional processes, it did not achieve normalization. Furthermore, contraindications such as hyperthyroidism, pregnancy, hypertonia, or substance abuse can prohibit treatment with methylphenidate (9, 17, 18). For instance, in a study investigating the efficacy of a combination of cognitive behavioral group psychotherapy, individual clinical management, and methylphenidate in a clinical sample, contraindications in about 20% of participants meant that they could not be treated with methylphenidate (14). Even if treatment with methylphenidate is possible, adverse side effects can occur. These most frequently include headaches, loss of appetite and weight, insomnia, internal unrest, and increased blood pressure and pulse (10). As a result, some patients prefer non-medical treatment.

Given the limitations of treatment with methylphenidate, it is worth considering alternative treatment approaches that target both the underlying neurobiological mechanisms and the psychosocial difficulties of patients with ADHD. From our point of view, this is best achieved by administering treatment from a neuropsychotherapeutic perspective. Such an approach incorporates neurobehavioral interventions to enhance the functioning of brain regions affected in ADHD and specific cognitive behavioral interventions. In mindfulness-based cognitive therapy (MBCT), conventional cognitive behavioral interventions are combined with mindfulness meditation, which can be understood as a form of mental training (19). Mindfulness signifies an open and alert state of mind. The person’s attention stays in the present moment, and sensations such as thoughts and feelings that arise are perceived and observed non-judgmentally (20). There is preliminary evidence that mindfulness meditation can improve the functioning of brain mechanisms underlying neuropsychological capacities impaired in ADHD, such as attention control and emotion regulation (19). We think that mindfulness meditation in patients with ADHD could help them to regulate brain functioning and thereby ameliorate their symptoms.

This paper aims to illustrate our concept of a neuropsychotherapeutic approach for ADHD in adulthood.

A search for trials on treatment, mindfulness, neuropsychotherapy, and psychotherapy in adult ADHD was conducted in the following bibliographic databases: PubMed, Embase, Medline, and Central (The Cochrane Central Register of Controlled Trials). The following terms were used: (ADHD OR (attention deficit) OR (attention deficit) OR hyperactivity*) AND (non-psychopharmacological treatment OR treatment OR therapy OR mindfulness OR psychotherapy OR neuropsychotherapy OR neuropsychology) AND (adult). Studies were selected and included according to their relevance for the subject.

## What is Neuropsychotherapy?

The concept of neuropsychotherapy represents the link between neuropsychology and psychotherapy. A neuropsychotherapeutic approach brings a neuroscientific perspective to therapeutic issues and aims to target underlying brain mechanisms that could be an obstacle to recovery in traditional therapy (21, 22).

We define “neuropsychotherapy” as an approach that integrates cognitive behavioral therapy with neurobehavioral treatment. Neurobehavioral treatment refers to behavioral interventions that deliberately target neuronal mechanisms associated with psychiatric disorders, in the same way as pharmacological or surgical treatments (22).

Since neuropsychotherapy targets neurobiological mechanisms, as well as observable symptoms or cognitive, behavioral manifestations of underlying neurobiological mechanisms, multiple therapeutic interventions are used. As in conventional psychotherapy, cognitive and behavioral interventions are employed for psychoeducative purposes and to bring about change in problematic cognitive and behavioral patterns. In addition, neuropsychological interventions are used. That is, affected neuronal structures are identified through neuropsychological assessment and neuroimaging. Subsequently, neurobehavioral interventions are employed to strengthen the functioning of those neuronal structures *via* concrete, intensive, and repetitive stimulation (21).

There is evidence that intensive and repetitive targeting of dysfunctional neuronal structures can ameliorate psychiatric disorders and improve brain functioning. In one study, patients with severe unipolar depression received neurobehavioral “cognitive control training” for 2 weeks, aimed at activating the prefrontal cortex, which it has been suggested plays an important role in depressive symptoms such as rumination. They were compared to a control group who received treatment as usual. Participants in the intervention group displayed a significantly greater decrease in depressive symptoms and rumination than participants in the control group. In addition, participants who received the cognitive control training showed normalization of brain functions targeted by the intervention (22).

Furthermore, it has been proposed that the success of cognitive behavioral therapy in treating anxiety disorders may be attributable to the modification of underlying, dysfunctional neuronal systems, as a result of the concrete, intense, and repetitive stimulation induced by exposure sessions (21). For instance, neuroimaging studies in OCD (obsessive compulsive disorder) samples (23, 24) revealed that exposure is associated with improved activity in brain areas involved in obsessive compulsive behavior.

It is recognized that, owing to neuronal plasticity, altered brain functioning can be modified by intense, prolonged, and regular therapeutic interventions (21, 22, 25), leading to improved psychological functioning. Therefore, the neuroscientifically informed implementation of neurobehavioral interventions in conventional psychotherapy appears to be a promising approach to improving treatment outcomes (22). It would appear particularly promising for treatment of a condition such as ADHD, which is known to be significantly related to structural, functional, and neurochemical brain abnormalities (26).

## Why Neuropsychotherapy in ADHD?

**Figure 1:**
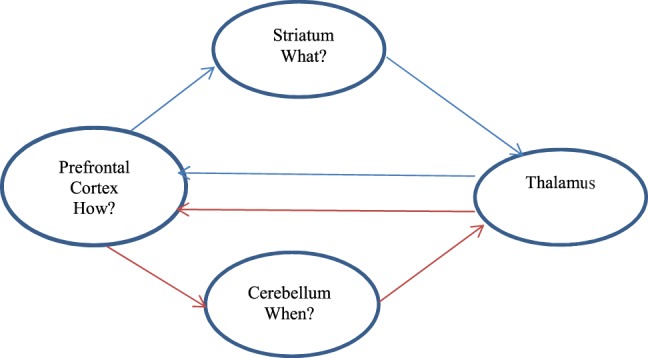
**Cerebellar pathways affected in ADHD**.

There is growing evidence that ADHD psychopathology is closely related to dysfunctions in multiple neuronal systems implicated in higher-level cognitive functions, as well as sensorimotor processes and the DMN (a brain network that is active in the resting state and inactive during task performance), which causes impairments in executive functioning (26), including in attentional processes such as sustained attention and set-shifting, impulse control, and working memory. ADHD symptoms are thought to reflect altered connectivity within and among several neural networks, rather than abnormal functioning of discrete, isolated brain regions (5). It has been suggested that mostly prefrontal–striatal–cerebellar circuits are impaired in ADHD in adulthood, specifically the prefrontal cortex, basal ganglia, and cerebellum are associated with ADHD (27, 28). The first of these neural circuits are frontostriatal loops, involved in response output control/response suppression, working memory, and response selection. Impairments are also found in frontocerebellar loops, responsible for the temporal information processing needed in timing and alerting the brain to new information. Finally, frontolimbic loops involved in avoidance conditioning and reinforcement learning are relevant to ADHD (27) (see Figure 1).

### Frontoparietal Network

A meta-analysis of neuroimaging studies of ADHD in adulthood revealed ADHD-related hypoactivation located in the frontoparietal network, which includes the lateral frontal pole, anterior cingulate cortex (ACC), dorsolateral prefrontal cortex (dlPFC), anterior PFC (aPFC), lateral cerebellum, anterior insula, and the caudate and inferior parietal lobe (5, 29). The frontoparietal network is known as the executive control circuit (30). It guides decision making by integrating external information with internal representations (5). It provides the flexibility to configure information processing when task demands change, and is involved in goal-directed executive processes (31). Hypoactivation in the frontoparietal network is consistent with executive dysfunction in ADHD in adulthood (26).

### Attentional Network

The functioning of the dorsal and ventral attentional networks, which are central parts of the attentional regulatory system, also seems to be impaired in ADHD (5, 32). The ventral attentional network includes the temporoparietal junction, the supramarginal gyrus, frontal operculum, and anterior insula (32). It is involved in attentional reorienting to relevant external stimuli and in interrupting ongoing activity when necessary. To prevent shifts of attention to irrelevant objects, suppression of this network is needed (33). It has been suggested that hyperactivation of the ventral attentional network may explain distractibility, which is a main symptom of ADHD (26). The dorsal attentional network, which is anchored by the intraparietal sulcus and the frontal eye fields, also shows ADHD-related abnormalities. The dorsal attentional network is associated with attention shifting and the control of spatial attention (32).

### Visual and Motor Network

Furthermore, impairments in the visual network have been proposed (34). This network includes the visual cortex and the middle temporal area complex, both of which are connected to the dorsal attentional network. The middle temporal area complex is also functionally correlated to frontal regions, as well as to primary visual areas (5, 35).

There is also evidence that the motor network (e.g., primary motor cortex, primary sensory cortex, putamen, thalamus, and cerebellum) may be affected in ADHD (5).

### Default-Mode Network

Attention deficit/hyperactivity disorder has also been conceptualized as a disorder of dysfunctional DMN activity. The DMN includes the medial prefrontal cortex (mPFC), ACC, and posterior cingulate cortex (PCC) (36). It has been suggested that in ADHD the inter-regulation between the DMN and networks activated during task performance (e.g., frontoparietal, ventral, or dorsal attentional networks) is disturbed. During task performance, DMN activity is typically suppressed. According to one hypothesis, in ADHD the DMN is hyperactive during task performance, which may cause the disruption in cognitive performance and fluctuation in attention that characterize the condition (26, 37). For example, the regulation of DMN activity by stimulant medication has been shown to improve cognitive performance in ADHD (38, 39).

Given these neurobiological functional impairments in ADHD, it seems rational to employ principles of neuroscience in treatment, which we discuss next.

## Mindfulness-Based Cognitive Therapy from a Neuropsychotherapeutic Perspective

A promising approach to improve the outcome of therapy in ADHD involves the neuropsychotherapeutic administration of meditation practice in MBCT. MBCT combines methods of cognitive behavioral therapy with mindfulness meditation. The treatment aims to provide the patient with an explanation for his symptoms, as well as information about ADHD. Behavioral interventions are designed to develop planning skills such as time management or problem solving. With cognitive methods, patients learn to identify and modify problematic thinking patterns (40). Besides cognitive behavioral interventions, patients engage in mindfulness meditation, which can be defined as a form of mental training that can improve neuropsychological deficits in ADHD, such as attention control and emotion regulation, by strengthening the function of brain regions believed to underlie these deficits (19).

It has been proposed that mindfulness meditation can help reduce mind wandering and distractibility in ADHD by improving the functioning of the DMN. For example, experienced meditators show reduced activation of the DMN during meditation and stronger functional connectivity of brain regions implicated in cognitive control and self-monitoring (41).

A further possible beneficial effect of mindfulness meditation in ADHD is that it teaches patients not to act out but rather to observe emotional states as temporary and passing events, thereby helping patients to improve their regulation of emotion. Emotion regulation refers to strategies that help to exert influence on the occurrence, experience, and expression of emotions (19, 42). Even though emotional dysregulation is not a core diagnostic feature of ADHD, it often contributes to considerable impairment (43–45). Neuroimaging studies have found neuroplastic changes in the structure and function of brain regions supporting emotion regulation (19).

The neurobiological mechanism of mindfulness meditation is currently not fully understood. It has been hypothesized that mindfulness meditation changes brain structure and function by myelinogenesis, synaptogenesis, dendritic branching, or adult neurogenesis (19). Furthermore, it seems possible that mindfulness meditation has a positive effect on neuronal preservation, restoration, and/or inhibition of apoptosis (46–48).

Besides these findings from neuroimaging studies, treatment studies have provided promising preliminary support for the feasibility and acceptability of mindfulness meditation in the treatment of ADHD. Recent studies indicate that mindfulness meditation training has ameliorating effects on ADHD symptoms and improves executive functioning, as well as emotion regulation (ISRCTN12722296 in preparation) (49, 50). Furthermore, participants show notable levels of compliance and report a high degree of satisfaction with the treatment (51). In addition, mindfulness meditation is a core component of a modified Dialectical Behavior Therapy group program for adults with ADHD. Mindfulness meditation seems to be well accepted by the patients and a very useful component of the program (52, 53) (see Table 1).

**Table 1:** **Evidence of changes after mindfulness meditation**.

Reference	Sample	Mean age	Duration	Results	
**Neuroimaging studies**					
(58)	Experienced mindfulness meditators/non-meditators	33.8	Meditators had 7.9 years of experience	Enhanced activation during meditation	Anterior cingulate cortex (ACC) (self-regulation of attention and emotion)
(47)	Students (integrative body-mind training vs. relaxation training)	21.5	5 days, 20 min a day	Greater activation of the ventral and/or rostral ACC during resting state after meditation	
(47)				Enhancement of the caudate nucleus and putamen during resting state following mindfulness meditation	Striatum (regulation of attention and emotion)
(41)	Experienced mindfulness meditators vs. healthy non-meditators	50.5	10 years experience	Reduced activation of the DMN during meditation	Default-mode network (brain network that is active in the resting state and inactive during task performance)
				Stronger functional connectivity of: posterior cingulate, dorsal anterior cingulate, and dorsolateral prefrontal cortices	
(59)	Healthy participants	26	6 weeks, 1066 min practice in total	Enhanced dorsolateral PFC activation during an emotional Stroop task	Prefrontal cortex (PFC) (attention and emotion)
(60)	Patients with general anxiety disorder vs. healthy controls	37.9	8-week program, once weekly, teacher-led group meetings plus one “day of mindfulness” in the sixth week of the course	Greater dorsolateral and dorsomedial PFC activation when participants were engaging in a mindful state while expecting to see negative emotional images	
**Clinical studies**					
(50)	Patients with ADHD	39.5	12 weekly sessions of 3 h MBCT, at-home practice	Reduced hyperactivity/impulsivity, as well as improved attention control	
(61)	Patients with ADHD	48.5	8 weekly sessions of 2.5 h of mindfulness training and daily at-home practice	Improvements in self-reported ADHD symptoms, anxiety and depression, improved performance on tasks measuring attention and cognitive inhibition	
(49)	Patients with ADHD	40.5	8 weekly sessions of 2.5 h of mindfulness training and daily at-home practice	Improved self-reported ADHD symptoms and improvement in executive functioning and in measured clinical ratings of ADHD symptoms	

Given this evidence, we conclude that engagement in mindfulness meditation is associated with functional changes in brain areas suggested to be impaired in adults with ADHD. In addition, patients readily accept mindfulness meditation. Thus, mindfulness meditation appears to be a promising neurobehavioral intervention, with several potential pathways to improving neuropsychological functioning in patients with ADHD.

## Limitations and Implications for Future Research

There is emerging evidence that mindfulness meditation ameliorates ADHD symptoms and may cause neuroplastic changes in brain regions impaired in ADHD. However, the study of the neurobiological mechanisms of mindfulness meditation and its beneficial effects in ADHD is still in its infancy. This allows only speculative statements about possible future treatment directions. The following limitations have to be considered, along with recommendations arising out of them.

Research is needed that uses larger sample sizes, active control conditions, and longitudinal, randomized research designs (19, 51).

To our knowledge, no neuroimaging study has investigated the effects of mindfulness meditation on the adult ADHD brain. Future research should aim to expand understanding of the neurobiological effects of mindfulness meditation in ADHD and to connect neuroscientific findings with behavioral data.

Furthermore, it has been suggested that ADHD is a heterogeneous condition, which probably includes different diagnostic subtypes (54), possibly caused by different neurobiological impairments. In addition, owing to the high comorbidity with other psychiatric disorders, common comorbid psychiatric disorders may rely on the same dysfunctional neuronal mechanisms as ADHD does. For example, ADHD and bipolar disorder show an overlap in diagnostic criteria, such as inattention and irritability (55). Also, a significantly higher prevalence of ADHD among relatives of persons with bipolar disorder and a significantly higher prevalence of bipolar I disorder among relatives of persons with ADHD has been reported (56). This co-occurrence may be associated with impairments in the same underlying neuronal mechanisms. Knowledge of these underlying neuronal mechanisms could help in developing a more specific assessment and classification of psychiatric disorders, as well as improved treatment interventions.

Another area for future research is investigation of the optimum amount of mindfulness meditation practice in ADHD. Neurobehavioral interventions are known to involve intense, prolonged, and regular stimulation of the targeted brain areas to effectively change neuronal structures (21). However, it is as yet not known how much mindfulness meditation is needed to evoke changes in neuroplasticity and psychological functioning in ADHD. For example, a study in a non-ADHD sample compared the effects of an 8-week mindfulness training course in a high- vs. low-practice group. The results indicate that high engagement in mindfulness (total time practice over 8 weeks: *M* = 11 h, SD = 7 h) compared to low engagement in mindfulness (total time practice over 8 weeks: *M* = 2.5 h, SD = 1 h) is associated with improved working memory control and improved positive affect (57).

Although studies indicate that mindfulness meditation has positive effects on ADHD in adulthood, the exact working mechanisms of mindfulness meditation are unclear. For example, patients may learn to relate and react more functionally to their thoughts thanks to mindful awareness of their cognitions, or to decenter from thoughts and view them as passing events that do not have to be acted upon. Furthermore, it is possible that mindfulness meditation causes changes in other areas of life, such as better self-care (e.g., improved diet, regular exercise), which could contribute to improved treatment outcomes.

## Conclusion

Attention-deficit/hyperactivity disorder, in adulthood is a serious mental condition with a strong neurobiological component that causes a wide range of impairments in affected individuals. Psychopharmacological medication is the first-line treatment for ADHD. However, not all patients respond well to it, and others have a preference for non-psychopharmacological treatment. Healthy psychological functioning in ADHD seems to rely greatly on the well-coordinated functioning of neuronal networks. There is promising preliminary evidence that mindfulness meditation employed as a neurobehavioral intervention in therapy can help ADHD patients to regulate impaired brain functioning and thereby improve self-regulation of attention and emotion control.

## Author Contributions

KB: literature research and writing, AL: literature research and writing, AP: literature research and writing, supervision.

## Conflict of Interest Statement

AP (MD) declares that she has served on advisory boards, given lectures, performed phase three studies, or received travel grants within the last 3 years from Eli Lilly and Co., Lundbeck, MEDICE Arzneimittel Pütter GmbH and Co. KG, Novartis, Servier, and Shire; and has authored books and articles on psychotherapy published by Elsevier, Hogrefe, Schattauer, Kohlhammer, Karger, and Springer. KB declares that the research was conducted in the absence of any commercial or financial relationships that could be construed as a potential conflict of interest. AL (MD) declares that she received travel grants within the last year from MEDICE Arzneimittel Pütter GmbH and Co. KG.
